# Quantum mechanics and 3D-QSAR studies on thienopyridine analogues: inhibitors of IKKβ

**DOI:** 10.1016/j.heliyon.2020.e04125

**Published:** 2020-06-11

**Authors:** Zaheer Ul-Haq, Alamgir Khan, Sajda Ashraf, Alejandro Morales-Bayuelo

**Affiliations:** a Dr. Panjwani Center for Molecular Medicine and Drug Research, International Center for Chemical and Biological Sciences, University of Karachi, Karachi 75270, Pakistan; b Grupo de Investigaciones Básicas y Clínicas de la Universidad del Sinú (GIBACUS), Escuela de Medicina, Universidad del Sinú, Seccional Cartagena de Indias, Colombia

**Keywords:** Pharmaceutical chemistry, Theoretical chemistry, Inhibitors of IKKβ, Thienopyridine analogues, Quantum mechanics, 3D-QSAR Studies, Chemical reactivity descriptors

## Abstract

Inhibitor of kappa B kinase subunit β (IKKβ) is a main regulator of nuclear factor kappa B (NF-κB) and has received considerable attention as an attractive therapeutic target for the treatment of lung cancer or other inflammatory disease. A group of diversified thienopyridine derivatives exhibited a wide range of biological activity was used to investigate its structural requirements by using DFT and 3D-Quantitative structure activity relationship. Comparative molecular field analysis (CoMFA) and comparative molecular similarity indices analysis (CoMSIA) were established using the experimental activity of thienopyridine derivatives. The cross-validation coefficient (q^2^) values for CoMFA and CoMSIA are 0.671 and 0.647 respectively, were achieved, demonstrating high predictive capability of the model. The contour analysis indicate that presence of hydrophobic and electrostatic field is highly desirable for biological activity. The results indicate that substitution of hydrophobic group with electron withdrawing effect at R^4^ and R^6^ position have more possibility to increase the biological activity of thienopyridine derivatives. Subsequently molecular docking and DFT calculation were performed to assess the potency of the compounds.

## 1 Introduction

Nuclear Factor Kappa B (NF-κB), a highly conserved and chief inducible transcription factor that regulates multiple aspects of immune system [1]. It plays a crucial role in coordinating inflammatory responses by regulating the adhesive molecules chemokines, vascular endothelial growth factor (VEGF), interleukin IL-1, interleukin IL-6, cyclooxygenase (COX)-2, matrix metallo proteinases (MMPs), 5-lipooxygenase (5-LOX) and tumor necrosis factor (TNF) [2]. As NF-κB is responsible for broad and diverse biological functions, NF-κB regulation is paramount to appropriate tissue allostasis [3]. The aberrant activation of NF-ĸB signaling results in several diseases, such as sensitivity to infections, cancers and autoimmunity [4, 5, 6]. Till to date around 15 pathways have been reported that are known to activate NF-κB. Among them canonical (classical) and noncanonical (alternative) are the most common pathways [7]. In canonical pathway, IKK is a trimeric complex composed of two catalytic sub-units IKKα, IKKβ and one regulatory subunit IKKγ. However, IKKβ is the convergence point for NF-κB signaling pathways. Inhibitor of nuclear factor kappa B kinase subunit beta (IKKβ) has been documented as an impotent kinase in NF-κB signaling. Phosphorylation of two serine amino acids adjacent to each other in IκB (Ser32/Ser36) is mediated by IKKβ which causes the ubiquitylation and consequently proteasomal degradation of IκB, resulting in NF-κB release [8, 9, 10]. Considering this, IKKβ is an important kinase for NF-κB signalling in response to inflammatory stimuli [11]. The vital role of IKKβ proposed that inhibition of this enzyme would be a promising strategy to treat cancers and other inflammatory diseases [12, 13]. As a result, drug-discovery efforts have identified several small molecules reported against the IKKs that are selective for IKKβ over IKKα. At present reported IKKβ inhibitors are characterized in to four different classes, including ATP analogues that are competitive inhibitors of ATP (Bay11-7082, MLN-120B, BI605906 and TPCA-1), allosteric inhibitors (BMS-345541), some natural products (ainsliadimer A, wedelactone) and thiol reactive compounds interacting with vital cysteine residues in IKKβ (berberine, nimbolide). Unfortunately, only a few of compounds are tested in phase I/II clinical trials. Three IKKβ inhibitors EB-1627, EB-1627 and IMD-1041 have been reported in different phases of clinical studies, but due to selectivity issue none of them have approved in phase III [14, 15]. Thus, new approaches for the discovery of IKKβ inhibitor require to be explored.

The Three-Dimensional Quantitative Structure Activity Relationship (3D-QSAR) modeling [16] is an *in silico* approach used by medicinal chemists to design new drugs [17]. The subject of present study is twofold, one is to develop the 3D-QSAR model for a set of 46 thienopyridine analogues [18, 19] with known biological activities and another is to explore the expediency of conceptual DFT quantities [20]. Molecular docking [21] was also employed in this method to find out the binding modes and active conformations of the compounds. The 3D-QSAR model was developed to explore the important structural features of thienopyridine analogues influencing the ligand-protein interaction by examining the biological activity of the compounds. To understand the complexity of 3D-QSAR results, chemical reactivity descriptors and molecular quantum similarity approach within the context of conceptual DFT were also performed to understand the substitution effect. The outcomes of present study are expected to provide the key structural features contributing in the binding mechanism and designing of novel and potential IKKβ inhibitors.

## 2 Material and methods

### 2.1 Curation of dataset

The data set was manually curated by selected 46 compounds of thienopyridine derivatives [18, 19, 32] (Table 1). The IC_50_ value of experimental activity of the dataset as a dependent variable transformed to its positive logarithmic scale by applying the equation: (pIC_50_ = −log IC_50_). The range of the pIC_50_ value from 4.77 to 7.38 log units, provided comprehensive and a homogenous data for 3D-QSAR modeling. The dataset was distributed into two set, training (35 compounds) and test set (11 compounds). Finally, the chemical diversity and activity distribution of the data set were analysed by CoMFA [33, 34] and CoMSIA [35] methods implemented in SYBYL7.3 [36].

**Table 1:** Selected 46 thienopyridine compounds for 3D quantitative structure activity relationship, changing at R^4^ and R^6^ position.

Compounds	R^4^	R^6^	IC_50_ (μM)	pIC_50_
1	H	Me	12.7	4.89
2	Me	Me	2.7	5.56
3	Pr	Me	1.3	5.88
4∗	Bu	Me	2.5	5.60
5	CH_2_OH	Me	13.5	4.86
6	Ome	H	4.7	5.32
7	Oet	H	2.0	5.69
8	Opr	H	2.2	5.65
9∗		H	10.8	4.96
10		Me	1.1	5.95
11		Me	16.9	4.77
12		Me	2.0	5.69
13		Me	6.0	5.22
14		Me	7.41	5.13
15		Me	8.50	5.07
16		Me	14.0	4.85
17		Me	1.30	5.88
18	HO(CH_2_)_2_O	Me	6.60	5.18
19	HO(CH_2_)_3_O	Me	5.10	5.29
20	HO(CH_2_)_2_	Me	5.80	5.23
21	H_2_N(CH_2_)_2_	Me	2.90	5.53
22		Me	2.41	5.61
23		Me	2.52	5.60
24		Me	2.41	5.61
25		Me	0.75	6.12
26		Me	0.68	6.16
27		n-Pr	0.57	6.24
28∗		n-Pr	0.28	6.55
29∗		n-Pr	0.12	6.92
30		n-Pr	0.68	6.16
31		n-Pr	0.04	7.38
32∗		n-Pr	0.12	6.92
33		n-Pr	0.68	6.16
34		n-Pr	0.52	6.28
35		n-Pr	2.01	5.69
36		n-Pr	1.32	5.88
37∗		n-Pr	0.15	6.82
38		n-Pr	7.81	5.10
39∗		n-Pr	0.098	7.00
40		n-Pr	1.45	5.83
41		n-Pr	1.95	5.70
42		n-Pr	0.59	6.22
43∗		n-Pr	0.50	6.30
44∗		n-Pr	0.07	7.14
45		n-Pr	0.63	6.20
46∗		n-Pr	0.14	6.85

∗ Selected compounds for Molecular Quantum set.

### 2.2 Molecular alignment

**Figure 1:**
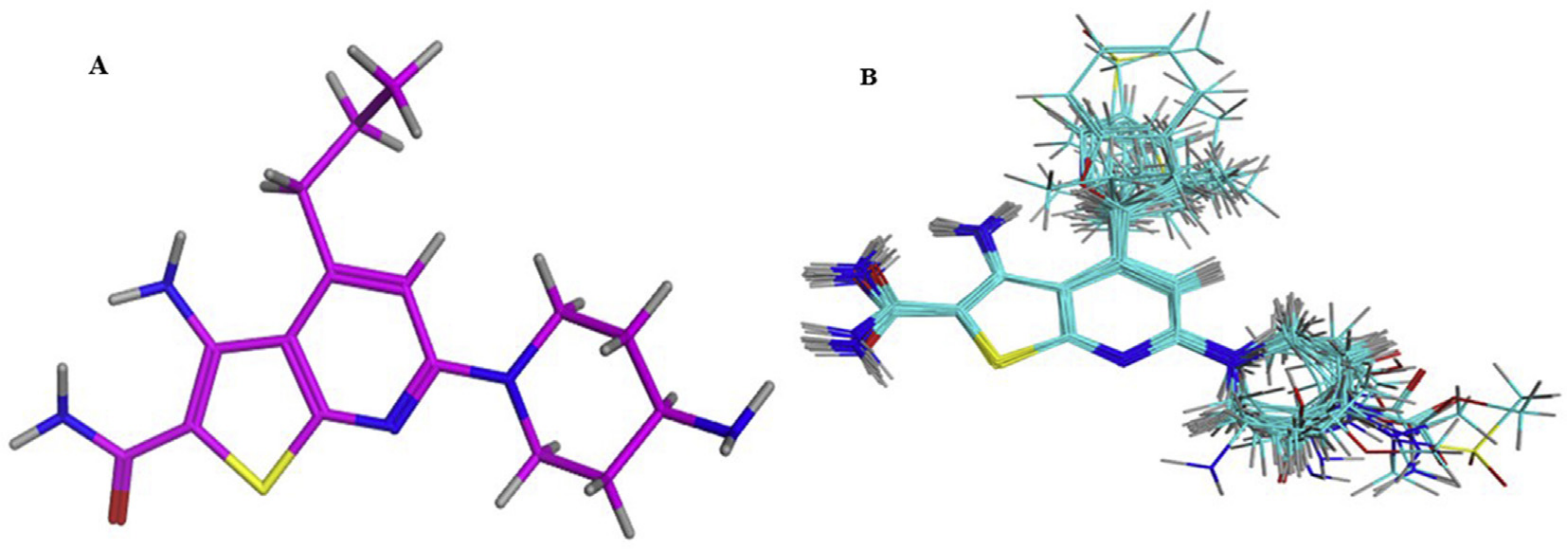
Molecular alignment (A) most active compound 31 among the dataset. (B) Alignment of 46 Thienopyridine analogues.

Molecular alignment was performed by flexible alignment application of MOE [37] to deduce the structural requirement of biological activity. The method accepts a collection of small molecules with three-dimensional coordinates to generate a collection of alignments. To quantify the alignment, score was assigned to each alignment in term of overlap molecular features and internal strain. In this work, top scored dock conformation of most active compound 31 was taken as a reference. FlexAlign generated random poses of the source molecule superposed onto the target molecule and optimized the alignment score S, while keeping the molecules rigid. Figure 1 shows the alignment of the selected data set.

### 2.3 CoMFA and CoMSIA setup

In 3D-QSAR, CoMFA is an alignment dependent descriptor method, used to correlate the structures to their experimental activity. The molecular interaction fields describe the structure by computing the interaction of a probe atom or molecule with the aligned conformation of the compounds at different points of 3D grid box. Conventional CoMFA technique was used to calculate steric and electrostatics properties on the basis of atom-centered monopoles, Coulombic and Lennard-Jones potentials [38].

For CoMSIA indices method, three additional fields (i.e. Hydrogen Bond Acceptor (HBA), Hydrogen Bond Donor (HBD) and Hydrophobic (H)), were added to the CoMFA to describe the molecular structure. In general, the accuracy and quality of these fields affect the exploratory power of 3D-QSAR model.

### 2.4 Partial least square (PLS) analysis

PLS regression algorithm [39] was used to build a direct correlation between interaction fields of CoMFA and CoMSIA to the pIC_50_ values. This correlation explains the changes in molecular descriptors with respect to the biological activity of the compounds. To obtain the cross-validated coefficient (q^2^) for CoMFA and CoMSIA, Leave-one-out (LOO) method was used, in which each compound was excluded from the database and the rest data used to build model to predict the activity. Cross-Validation method was used to determine the optimal number of components (ONC) which was evaluated by high q^2^ correlation coefficient, the fisher test (F value) and the small standard error of prediction (SEP). PLS analysis was performed multiple times for the entire modeling using ONC by keeping the grid spacing of 0.6 Å with 2.00 kcal/mol of column filtering to speed up the analysis and reduce the noise.

### 2.5 Docking analysis

Molecular docking is one of the most popular technique used in structure based drug design due to its ability to forecast with high grade of accuracy [40]. The IKKβ crystal structure was retrieved from Protein Data Bank with PDB ID: 4KIK [41]. The structure was prepared by using protein preparation option in MOE. The structure was further optimized by correcting atom type, bond order, formal charge and addition of corresponding hydrogen atoms. AMBER10:EHT force field [42] in MOE was used to apply partial charges followed by energy minimization. Molecular modeling of thienopyridine derivatives was performed SYBYL 7.3 software package [36]. All compounds were built using sketch module in SYBYL7.3. Compounds were minimized by MMFF94 force field [43] based on lowest energy conformation. The threshold of energy convergence gradient was set to 2.1 kJ/(mol nm) while 1000 number of iterations were used. Before docking, the rationality of the docking protocol and software was validated by re-docking of the reference compound to the active site of IKKβ using default parameters. The RMSD value of the predicted pose with reference to cognate ligand was <2.0 Å validated the selection of the docking parameters.

### 2.6 Chemical reactivity analysis

For the development of molecular quantum set 10 compounds involving structural and biological activity diversity were selected as shown in Table 2. All quantum calculations were carried out at the B3LYP/6-31G (d,p) level of theory [22] using GAUSSIAN 09 program package [23]. The information about the energies of frontier molecular orbitals was obtained by single point energy computation at the B3LYP/6-31G (d,p) level.

**Table 2:** Global chemical reactivity descriptors. Chemical potential (μ), hardness (η), and electrophillicity (ω) in eV and softness (S) in eV^−1^.

Compound	C. Potential (μ)	hardness (η)	Softness (S = 1/η)	electrophilicity (ω)
4	-3.7843	3.8036	0.2629	1.8825
9	-3.8531	3.7443	0.2670	1.9826
28	-2.8058	1.8096	0.5526	2.1752
29	-2.7818	1.7660	0.5662	2.1910
32	-2.4749	1.6359	0.6112	1.8720
37	-3.5558	3.3342	0.2999	1.8961
39	-2.8369	1.6847	0.5936	2.3887
43	-3.5617	3.6267	0.2757	1.7489
44	-3.5320	3.5277	0.2835	1.7682
46	-3.6697	3.6256	0.2758	1.8572

In this study the descriptors of chemical reactivity supported by DFT [24] like hardness (η) [25, 26], chemical potential (μ) [27, 28] and electrophilicity (ω) [24, 29]. Moreover, local reactivity descriptors of Fukui function ($f_{k}^{x}$) [30, 31] condensed to atoms were used to get insight into the stabilization process of active site.

The global chemical reactivity descriptor (μ) calculate the escaping tendency of electron from the electronic cloud. Global hardness or Eta (η) calculate the resistance towards polarization or deformation of the electronic cloud (atoms, ions and molecules) under chemical reaction with minor perturbation. The electronic behavior of the system was characterized by electrophilic index (ω) that estimate the ability of compound to cause transfer of an electron. These descriptors can be computed by the following equations:

(1) $$\mu \;={\left(\frac{\partial E}{\partial N}\right)}_{v\left(r\right)}\;$$

(2) $$\eta \;=\;{\left(\frac{\partial^{2}E}{\partial N^{2}}\right)}_{v\left(r\right)}$$

where v(r). *ω* is defined as

(3) $$\omega =\frac{\mu^{2}}{2\eta }=\frac{\chi^{2}}{2\eta }$$

Utilizing the approximations of finite difference in Eqs. (1) and (2), μand η can be stated as:

(4) $$\mu =-\frac{I+A}{2}$$

and

(5) η=I−A

Here, *A* and *I* are the electron affinity and ionization potential, respectively. *A* and *I* were computed by Koopmans' theorem.

(6) Where, *I* = -*E*_HOMO_

(7) and *A* = -*E*_LUMO_

The Fukui functions $f_{k}^{x}$(*x* = +, -) as local chemical reactivity descriptors were computed as follows:

(8a) $$f_{k}^{+}=q_{k}\left(N+1\right)-q_{k}\left(N\right)\;\text{for nucleophilic attack}$$

(8b) $$f_{k}^{-}=q_{k}\left(N\right)-q_{k}\left(N-1\right)\;\text{for electrophilic attack}$$

The electron population of compound refers as *q*_*k*_ at *k*^*th*^ atomic site. In this study, natural population analysis (NPA) method was used to calculate atomic charges.

## 3 Results and discussions

### 3.1 Molecular docking

**Figure 2:**
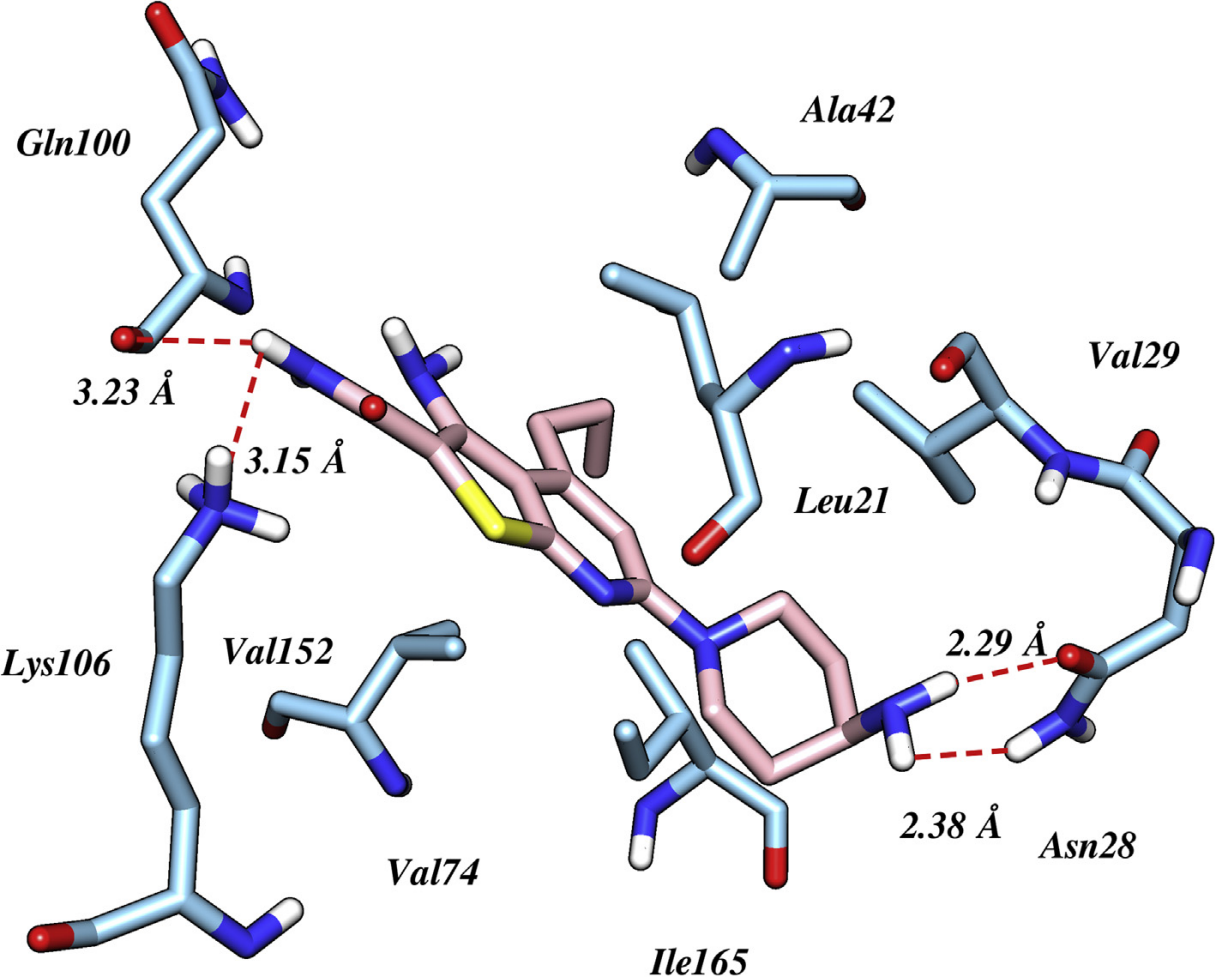
The docked pose of cognate ligand in the active sites of IKKβ with the interactions with crucial residues along their interaction distance in Armstrong.

Docking simulation helps to evaluate the binding mechanism of thienopyridine derivatives by estimating the binding energies and intermolecular distance between the interacting residues to the compounds. Based on binding energy and interactions obtained from docking analysis, the best-scored conformations were selected for the generation of 3D-QSAR model. Docking results indicate that the investigated compounds bind to the active site of the kinase domain of IKKβ located at the hinge region connecting C-lobe and N-lobe. The binding mode of compound 31 (IC_50_ = 0.041 μM) was selected for the descriptive analysis, as it was the most representative member of the series. The 4-amino-piperidyl ring of compound 31 involved in making two strong hydrogen bond interactions with the side chain of Asn28 at a distance of 2.38 and 2.29 Å respectively. Additionally, two more hydrogen bonds were formed between NH of carboxamide to the carbonyl and amino group of Gln100 and Lys106 at a distance of 3.23 and 3.13 Å respectively. The presence of these additional hydrogen bonds describes the high binding affinity of compound 31 (Figure 2) as compared to other derivatives of the series. Further, Kalia and Kukol also stated that potential IKKβ inhibitors should deeply buried in the hydrophobic groove of the ATP pocket. In our study most active compound 31 also accommodated in the same hydrophobic cavity by making promising interactions Leu21, Val29, Ala42, Val74, Val152 and Ile165. However, compound 16 (least active) did not possess all these interactions with the crucial residue of the binding site.

### 3.2 CoMFA and CoMSIA results

**Figure 3:**
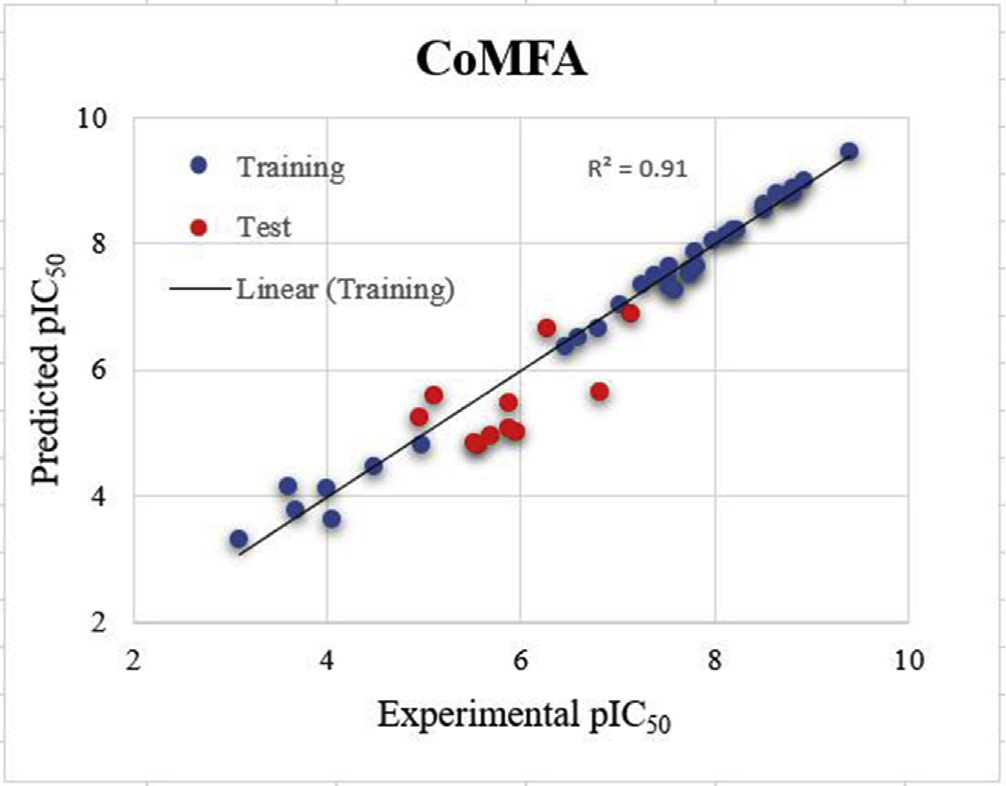
Experimental pIC_50_ values were plotted against (x-axis) against the predicted pIC_50_ values (y-axis) from the CoMFA model of structure-based 3D-QSAR.

**Figure 4:**
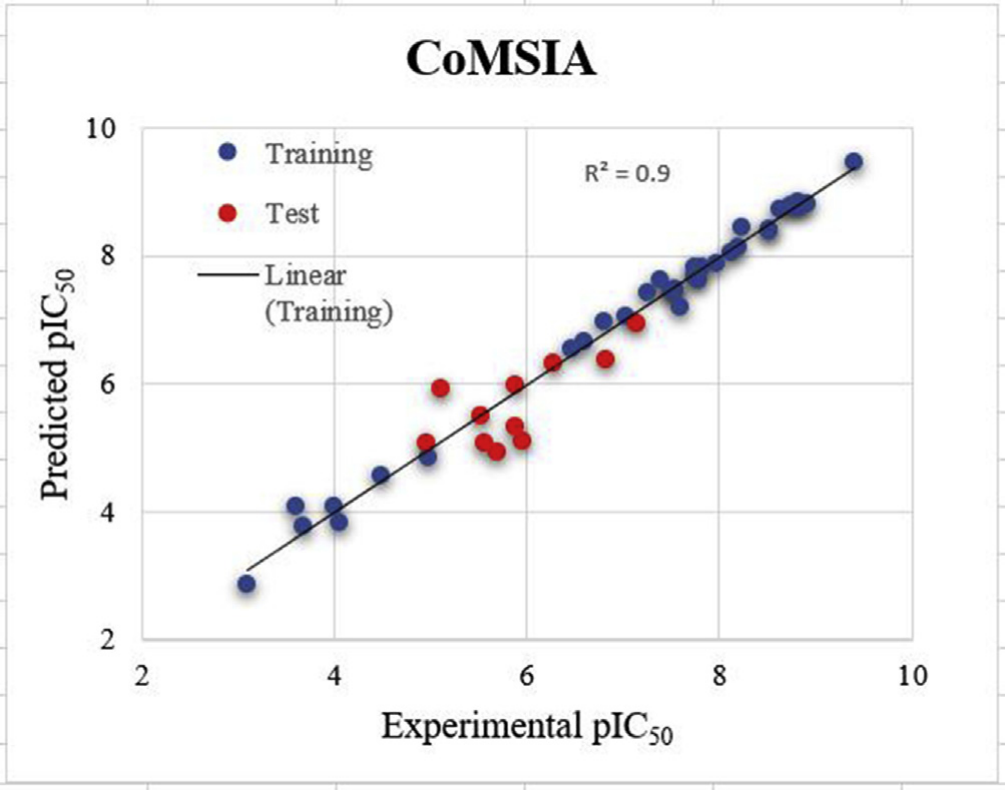
Experimental pIC_50_ values were plotted against (x-axis) against the predicted pIC_50_ values (y-axis) from the CoMSIA model of structure-based 3D-QSAR.

CoMFA and CoMSIA models were generated of all 46 thienopyridine compounds with bioactivity value range 4.77–7.38 log units. The statistical parameters of the developed 3D-QSAR models are summarized in Tables 3 and 4. The developed CoMFA model on IKKβ inhibitors produced a cross-validated coefficient q^2^ of 0.671 with six optimal number of component (ONC), a non-cross validated coefficient (r^2^) of 0.989 with Standard Error of Estimation (SEE) of 0.077 and F value of 435.87 respectively. These statistical parameters validate the robustness of the model. By relating the corresponding field contributions of electrostatic and steric descriptors (Table 4), it was designated that electrostatic fields of thienopyridine derivatives would have a comparatively higher impact (51%) on its inhibitory activity to IKKβ than steric contribution (49%). CoMSIA models were produced using five descriptors, i.e. steric field (S), electrostatic field (E), hydrophobic field (H), a hydrogen bond donor (D), and a hydrogen bond Acceptor (A), based on IKKβ inhibitors. The corresponding field contributions were 13%, 25%, 22%, 20% and 20%, for steric, electrostatic, hydrophobic field, hydrogen bond donor, and hydrogen bond acceptor field, respectively. The CoMSIA model provided a cross-validated coefficient q^2^ of 0.646, a non-cross validated coefficient of 0.950, a standard error of prediction value of 0.162, and an F value of 143.599 with four optimal number of components. The results recommended that the electrostatic and hydrogen bond acceptor field show greater contributions in the binding of IKKβ inhibitors. The experimental and predicted pIC_50_ values for the training set and the test set are listed in Table 5. Figures 3 and 4 illustrate the linear regression between experimental and predicted pIC_50_ values of training and test set compounds for CoMFA and CoMSIA models, respectively.

**Table 3:** Statistical parameters for structure based 3D-QSAR based on CoMFA model.

Item	CoMFA
Optimum Number of Components	6
Cross-Validated Coefficient (q^2^)	0.671
Non-Cross-Validated Validation Coefficient (r^2^)	0.989
Standard Error of Estimate	0.077
Fischer Statistic Value	435.87

Fraction of Field Contribution in %	
Steric Field	49 %
Electrostatic Field	51 %

**Table 4:** Statistical parameters for structure based 3D-QSAR based on CoMSIA model.

Items	CoMSIA
Optimum Number of Components	4
Cross-Validated Coefficient (q^2^)	0.646
Non-Cross Validated Validation Coefficient (r^2^)	0.950
Standard Error of Estimate	0.162
Fischer Statistic Value	143.599

Fraction of Field Contribution in %	
Steric Field	13 %
Electrostatic Field	25 %
Hydrophobic Field	22 %
Hydrogen Bond Donor Field	20 %
Hydrogen Bond Acceptor Field	20 %

**Table 5:** Experimental activity versus predicted activity pIC_50_ values of Thienopyridine analogues with functional data (residual) of training and test set for structure based 3D-QSAR.

No of	pIC_50_	CoMFA	CoMFA	CoMSIA	CoMSIA
Compound	Activity	Prediction	Functional Data	Prediction	Functional Data
1	4.89	4.81	0.08	4.92	0.03
2∗	5.56	4.81	0.75	5.07	-0.49
3∗	5.88	5.09	0.79	5.34	-0.54
4	5.60	5.63	-0.03	5.62	0.02
5	4.86	4.93	-0.07	4.83	-0.03
6	5.32	5.40	-0.08	5.19	-0.13
7	5.69	5.68	0.01	5.44	-0.25
8	5.65	5.66	-0.01	5.57	-0.08
9∗	4.96	5.26	-0.30	5.07	0.11
10∗	5.95	5.01	0.94	5.11	-0.84
11	4.77	4.88	-0.11	4.72	-0.05
12∗	5.69	4.95	0.74	4.93	-0.76
13	5.22	5.20	0.02	5.18	-0.04
14	5.13	5.09	0.04	5.04	-0.09
15	5.07	5.05	0.02	5.02	-0.05
16	4.85	4.80	0.05	5.00	0.15
17	5.88	5.85	0.03	5.99	0.11
18	5.18	5.22	-0.04	5.36	0.18
19	5.29	5.34	-0.05	5.48	0.19
20	5.23	5.16	0.07	5.20	-0.03
21∗	5.53	4.85	0.68	5.50	-0.03
22	5.61	5.73	-0.12	5.77	0.16
23	5.60	5.70	-0.10	5.69	0.09
24	5.61	5.66	-0.05	5.58	-0.03
25	6.12	6.07	0.05	5.99	-0.13
26	6.16	6.00	0.16	6.28	0.12
27	6.24	6.24	0.00	5.98	-0.26
28	6.55	6.54	0.01	6.28	-0.27
29	6.92	6.99	-0.07	6.80	-0.12
30	6.16	6.17	-0.01	6.02	-0.14
31	7.38	7.20	0.18	7.25	-0.13
32	6.92	7.00	-0.08	7.01	0.09
33	6.16	6.06	0.10	6.33	0.17
34∗	6.28	6.66	-0.38	6.32	0.04
35	5.69	5.60	0.09	6.07	0.38
36∗	5.88	5.49	0.39	6.00	0.12
37∗	6.82	5.65	1.17	6.39	-0.43
38∗	5.10	5.59	-0.49	5.92	0.82
39	7.00	7.04	-0.04	6.94	-0.06
40	5.83	5.87	-0.04	5.83	0.00
41	5.70	5.64	0.06	5.72	0.02
42	6.22	6.15	0.07	6.52	0.30
43	6.30	6.28	0.02	6.10	-0.20
44∗	7.14	6.88	0.26	6.96	-0.18
45	6.20	6.26	-0.06	6.15	-0.05
46	6.85	6.89	-0.04	6.82	-0.03

∗ Test set compounds.

### 3.3 CoMFA contour analysis

To visualize the field effect in three-dimensional space, the resultant contour maps that were generated by CoMFA and CoMSIA were analysed by overlaying the most active and inactive compound of the series. The contour maps around the molecules provide valuable insight into the nature and position of the chemical features that required for the biological activity.

**Figure 5:**
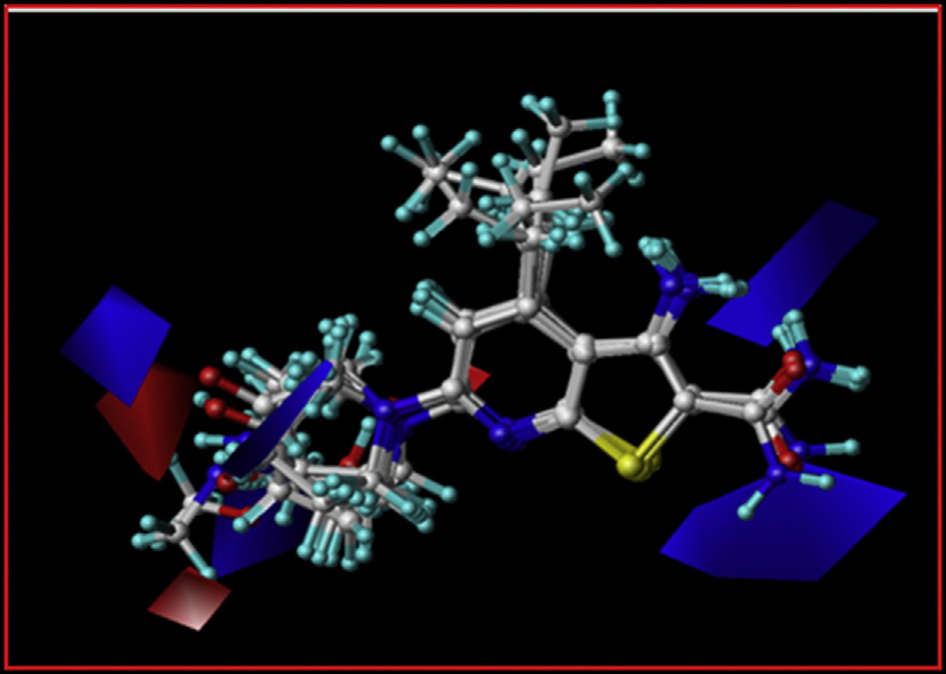
The electrostatic contours of the selected groups of compounds, the blue contour shows favorable electrostatic regions. While, the red contours depict regions where charged group are disfavored. The green and red arrows depict R^6^ and R^4^ respectively.

**Figure 6:**
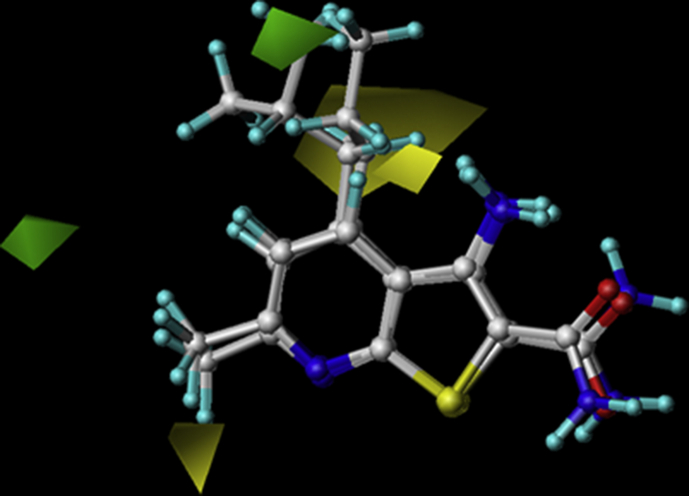
The steric contours of the selected groups of compounds, the yellow contour shows favorable steric regions. While, the green contours depict regions where bulky group are disfavored. The green and red arrows depict R^6^ and R^4^ respective.

In the CoMFA, sterically favorable contour map presented by green colour while yellow represents the opposite. While blue and red contour represents the region where electropositive and electronegative group are favoured and disfavoured respectively. The 4-aminopiperidyl group was encompassed by yellow contour, which indicate that any bulky substitution to this position could reduce the biological activity. This possibly explained that mono and di-methylation of NH_2_ group decrease the activity in case compound 32 and 33 verified this observation. Besides, a green contour adjacent to R^4^ position suggesting that presence of aliphatic chain at this position leads to enhance the inhibitory activity (Figure 5). For instance, compound 1 and 2, bearing hydrogen and methyl group show less activity. Blue areas near amide and amino-piperidyl group on C-2 and C-6 positions imply that positive substitution is favoured in these regions (Figure 6). This effect may be explained in case of compound 22, 25 and 26 in which polar group attached to the six-membered ring show modest improvement in activity. Compound 25 with 4-hydroxypiperidyl group show three-fold more potency than compound 22 with only piperidyl ring.

### 3.4 CoMSIA contour analysis

**Figure 7:**
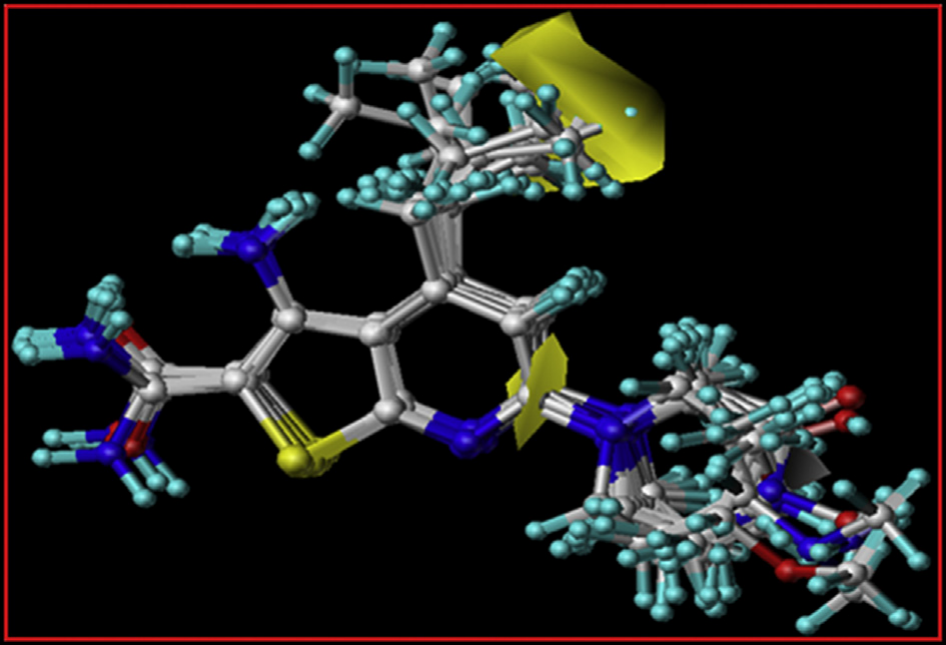
The hydrophobic contours of the selected groups of compounds, the yellow contour shows favorable hydrophobic regions. While, the white contours depict regions where hydrophobic groups are disfavored. The green and red arrows depict R^6^ and R^4^ respectively.

CoMSIA is an extension of CoMFA method and differ only in the implementation of three more fields including hydrophobic, hydrogen-bond donor and hydrogen-bond acceptor. The contour map for hydrophobic field presented in Figure 7. The white and yellow contours represent the areas where hydrophobic substitution have negative and positive effects on the overall activity. Yellow contours near the pyridine ring and the aliphatic propyl chain at R^4^ position indicating that hydrophobic substitution in these regions are favourable. This observation is consistent with the fact that compound 3 with propyl chain has more activity than compound 1 and 2 with hydrogen and methyl substitution. A white contour near amino piperidyl group reveal that hydrophobicity at NH2 does not help in increasing the activity. Hence, methylation of NH2 in compound 32 and 33 hold lower activity than compound 31 without methylation.

**Figure 8:**
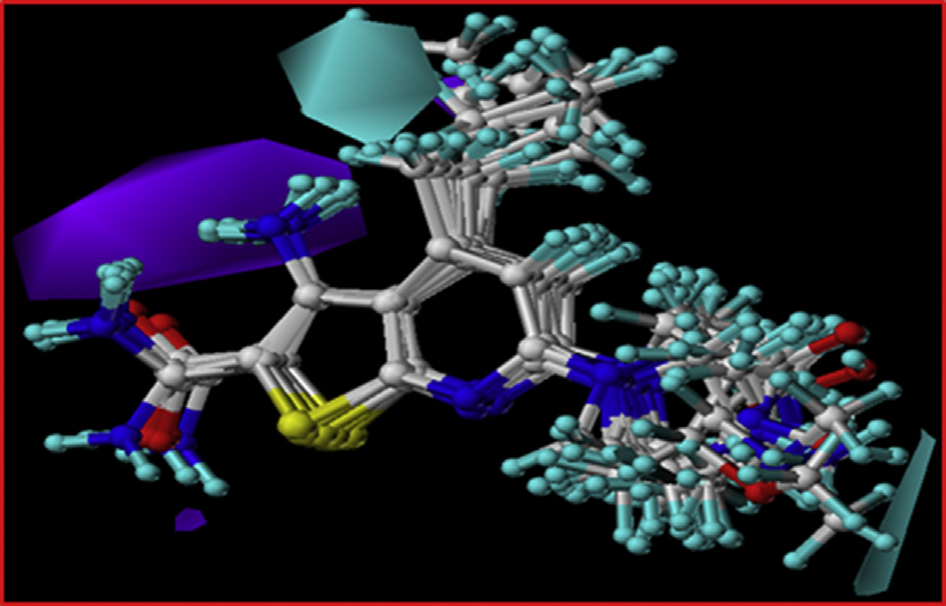
The Hydrogen bond donor contours of the selected groups of compounds, the cyan contour shows favorable hydrogen bond donor regions. While, the purple contours depict regions where hydrogen bond donating groups are disfavored. The green and red arrows depict R^6^ and R^4^ respectively.

**Figure 9:**
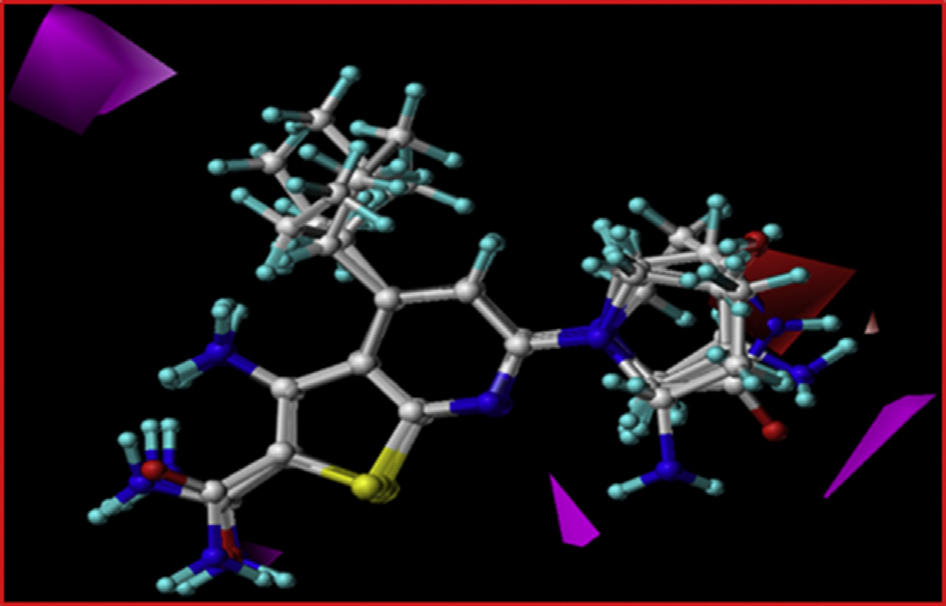
The Hydrogen bond acceptor contours of the selected groups of compounds, the magenta contour shows favorable hydrogen bond acceptor regions. While, the red contours depict regions where hydrogen bond accepting groups are disfavored. The green and red arrows depict R^6^ and R^4^ respectively.

The molecular depiction of the most active compound with their respective hydrogen bond donor and acceptor fields was provided in Figures 8 and 9. Cyan and purple contours designate the areas where substitution of donating group can increase or decrease the activity. While magenta and red contour specify the favour and disfavour region for hydrogen bond accepting group. A region where both cyan and red contour is present near the piperidyl ring suggesting that presence of donating group in this region can improve the activity. This may be due to two strong hydrogen bond interactions of 4-amino-piperidyl ring with crucial active site residue Asn28. Further, a magenta contour found near the R^4^ position indicate that presence of hydrogen bond acceptor group leads to increase the activity.

#### 3.4.1 Molecular quantum set analysis

Currently, there is a growing interest in the interpretation of the QSAR methods like 3D-QSAR from the Quantum Chemistry [44, 45, 46] point of view. Therefore, in this study the 3D-QSAR results were supported with DFT calculations using the most active compounds. The global chemical reactivity descriptors are shown in Table 2.

The compound with the higher C. potential is the compound 9 (μ=-3.8531 eV), it also has the higher hardness (η=3,8036 eV) and the lowest Softness (S = 0.2629 eV^−1^). The compound with higher electrophillicity is 39 (ω=2.3887 eV). The most active compound 44 (pIC_50_ = 7.14) has a chemical potential of (μ=-3.5320 eV), hardness (η=3.5277 eV), Softness (S = 0.2835 eV^−1^) and electrophillicity (ω=1.7682 eV). These values can be related with non-covalent interactions in the stabilization process on the active site. The compounds 28, 29, 32 and 39 have low hardness values and low softness values. Therefore, these compounds have most susceptibility to develop a retro-donor process on the active site. In this order of ideas, the global reactivity descriptors talk about the retro-donor process on the active site and give an idea about the stabilization.

**Figure 10:**
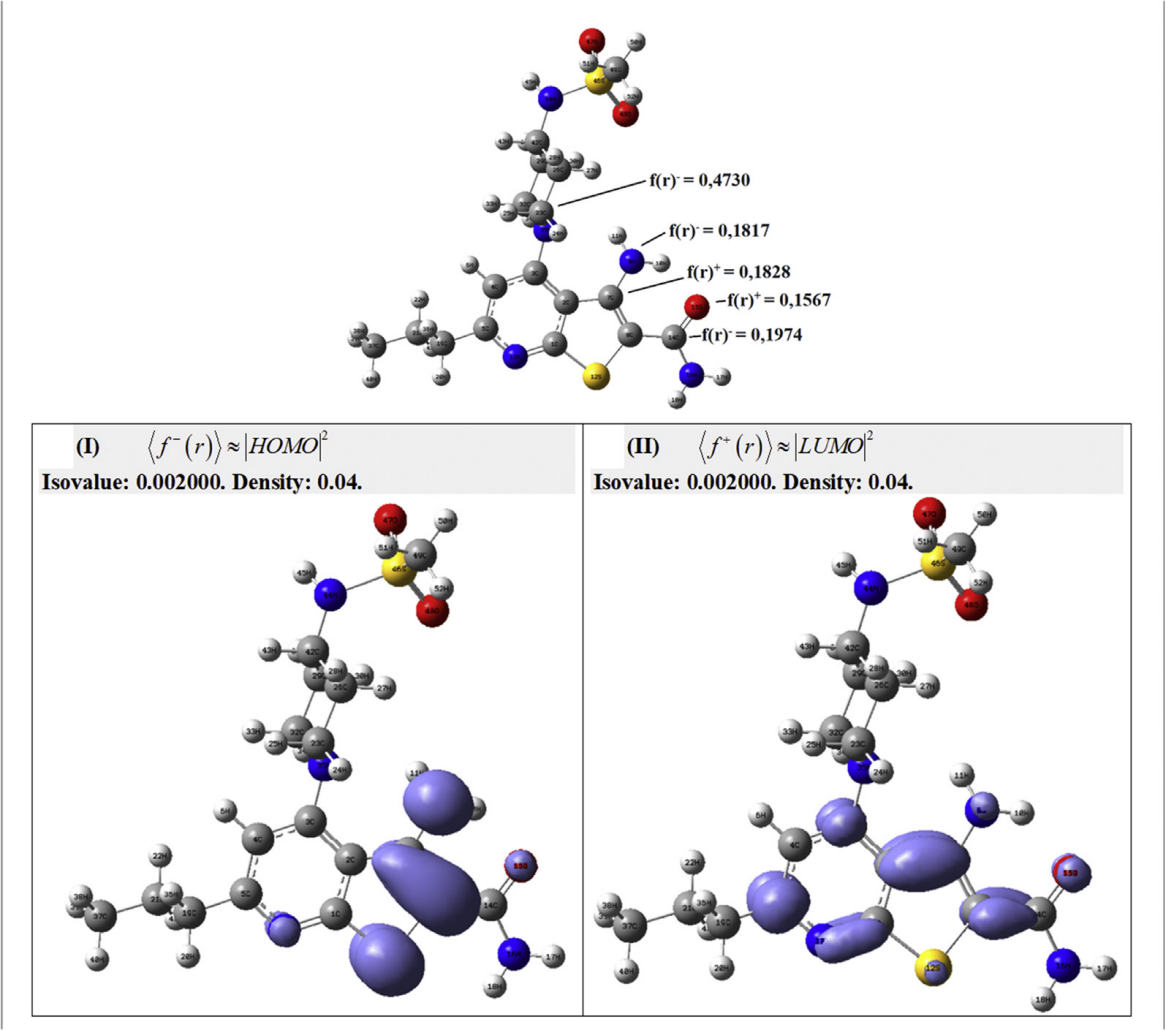
Fukui functions of compound 39 taken in Molecular Quantum set.

**Figure 11:**
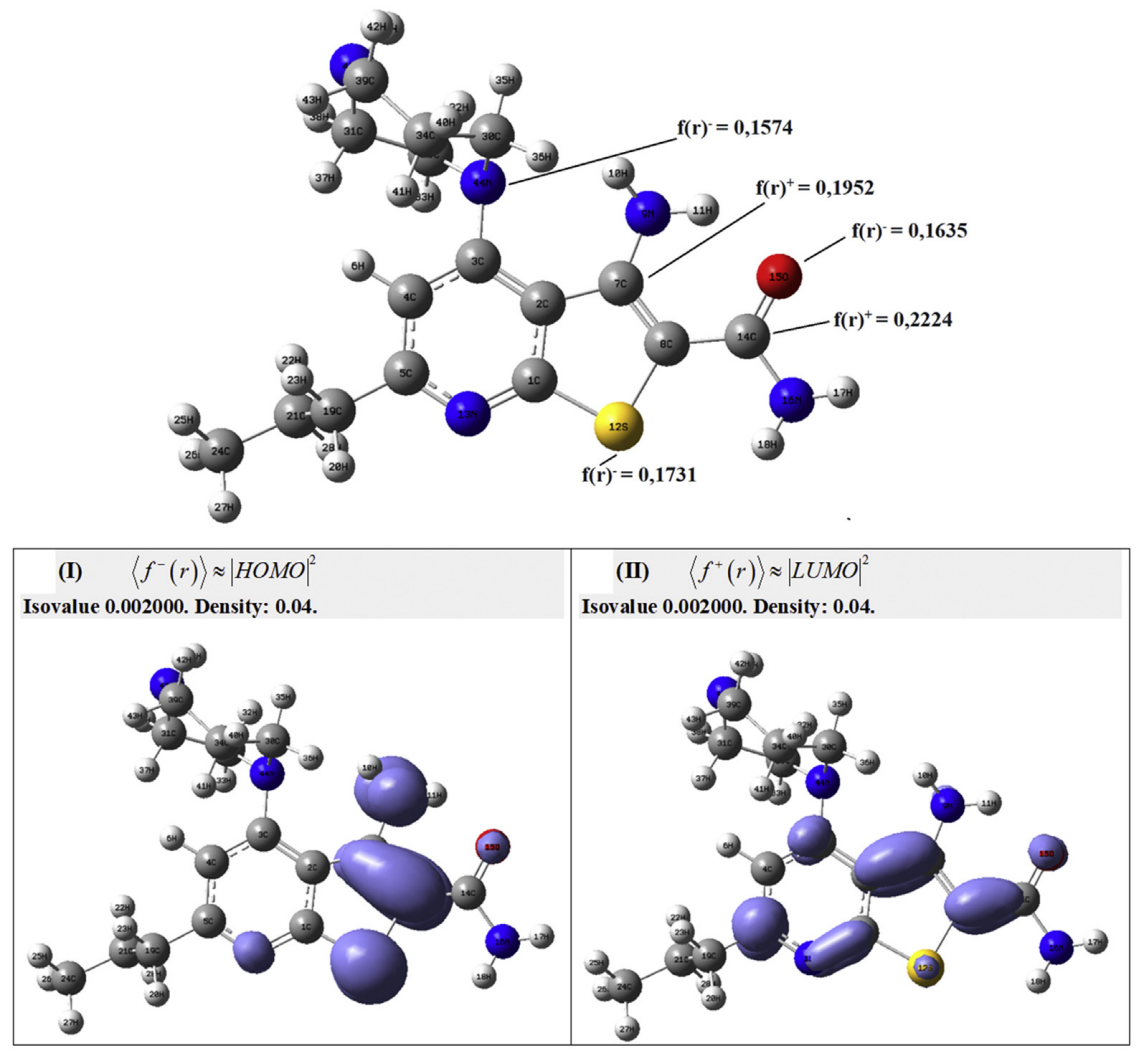
Fukui functions of compound 44 taken in Molecular Quantum set.

In the compound 39, the higher $f^{+}\left(r\right)$ is in the carbon atom 14C (0.1974), therefore, under this site are developed the most important nucleophilic attacks (Figure 10). The higher susceptibility to electrophilic attack $f^{-}\left(r\right)$ is in the carbon atom 30C (0.4730). To the compound 44 the higher $f^{+}\left(r\right)$ is in the carbon atom 14C (0.2224) and the most important site to electrophilic attack are in the carbon atom 7C (Figure 11). This local chemical reactivity shows the most important zones in the stabilization process through non-covalent interactions, these zones may be related with the maps from the CoMFA and CoMSIA results.

On the other hand, the removal of water molecules from the cavity and breaking of hydrogen bonds leads to an increase in entropy and is a driving force for the ligand, very effective for the compound 44 (pIC_50_ = 7.14) and much less for compounds 28, 29, 32 and 39. Thus, the ability to take part in the intermolecular interactions reflects the trend of chemical reactivity, which is based on the electronic properties in the thienopyridine derivatives. Such an analysis allows a fast prediction of the ability of the thienopyridine derivatives to bind with IKKβ as its inhibitor. For this reason, in Figures 10 and 11, we can see the isosurfaces for the most active compound 39 and 44, respectively: (a) Isosurfaces$\left\langle f^{-}\left(r\right)\right\rangle \approx {\left|HOMO\right|}^{2}$ and $\left\langle f^{+}\left(r\right)\right\rangle \approx {\left|LUMO\right|}^{2}$ for the compound 39. (b) Isosurfaces$\left\langle f^{-}\left(r\right)\right\rangle \approx {\left|HOMO\right|}^{2}$ and $\left\langle f^{+}\left(r\right)\right\rangle \approx {\left|LUMO\right|}^{2}$ for the compound 44. The isosurface $\left\langle f^{-}\left(r\right)\right\rangle \approx {\left|HOMO\right|}^{2}$is realted with the suceptibility that has a molecule to electrophilic attack and the isosurface $\left\langle f^{+}\left(r\right)\right\rangle \approx {\left|LUMO\right|}^{2}$ is related with the suceptibility that has a molecule to nucleophilic attack. In this order of ideas, in the most active compounds are presented the zones related with the stablization process into the cavity.

## 4 Conclusion

In conclusion, the current study deals with the development of receptor guided 3D-QSAR model on thienopyridine derivatives for exploring the key factors that are responsible for their inhibitory mechanism on IKKβ. The generated model exhibited good predictive power and satisfactory correlation between theory and experiment. The studied mechanism unravels the structural necessities at C4 and C6 position including polar functional groups with partial hydrophobic effect, while the methylation of NH in the pyrazinyl analogues could decrease the inhibitory activity. Molecular docking results indicated that thienopyridine derivatives interacting with ATP pocket in a same mode. Each compound displayed at least one hydrophillic interaction with binding site residues Asp28, Gln100 and Lys106 and some non-bonded interactions with residues of activation loop that stabilizes IKKβ interaction with thienopyridine derivatives. In addition, molecular quantum studies were performed to calculate the global reactivity (electrophilicity, hardness and chemical potential) and local reactivity descriptor (Fukui functions) to describe the reactive site of the molecules. Both type of descriptors was found to be effective in predicting the physicochemical properties of the molecule. The outcomes of the current study would provide additional insight for the design of potential and novel IKKβ inhibitors.

## Declarations

### Author contribution statement

Zaheer Ul-Haq: Conceived and designed the experiments; Contributed reagents, materials, analysis tools or data; Wrote the paper.

Alamgir Khan: Performed the experiments; Analyzed and interpreted the data; Wrote the paper.

Sajda Ashraf: Analyzed and interpreted the data.

Alejandro Morales-Bayuelo: Performed the experiments; Wrote the paper.

### Funding statement

This research did not receive any specific grant from funding agencies in the public, commercial, or not-for-profit sectors.

### Competing interest statement

The authors declare no conflict of interest.

### Additional information

No additional information is available for this paper.
